# Identification of Haptic Based Guiding Using Hard Reins

**DOI:** 10.1371/journal.pone.0132020

**Published:** 2015-07-22

**Authors:** Anuradha Ranasinghe, Prokar Dasgupta, Kaspar Althoefer, Thrishantha Nanayakkara

**Affiliations:** 1 Department of Informatics/ Center for Robotics Research, King’s College London, London, United Kingdom; 2 MRC Center for Transplantation, DTIMB & NIHR BRC, King’s College London, London, United Kingdom; University of Perugia, ITALY

## Abstract

This paper presents identifications of human-human interaction in which one person with limited auditory and visual perception of the environment (a follower) is guided by an agent with full perceptual capabilities (a guider) via a hard rein along a given path. We investigate several identifications of the interaction between the guider and the follower such as computational models that map states of the follower to actions of the guider and the computational basis of the guider to modulate the force on the rein in response to the trust level of the follower. Based on experimental identification systems on human demonstrations show that the guider and the follower experience learning for an optimal stable state-dependent novel 3^rd^ and 2^nd^ order auto-regressive predictive and reactive control policies respectively. By modeling the follower’s dynamics using a time varying virtual damped inertial system, we found that the coefficient of virtual damping is most appropriate to explain the trust level of the follower at any given time. Moreover, we present the stability of the extracted guiding policy when it was implemented on a planar 1-DoF robotic arm. Our findings provide a theoretical basis to design advanced human-robot interaction algorithms applicable to a variety of situations where a human requires the assistance of a robot to perceive the environment.

## Introduction

Robots have been used in urban search and rescue (USAR) for the last ten years [1]. Human-Robot Interaction (HRI) is a field to study dedicated to understanding, designing, and evaluating robotic systems for use by or in interaction with humans [2]. The need for advanced HRI algorithms that are responsive to real time variations of the physical and psychological states of human users in uncalibrated environments has been felt in many applications like fire-fighting, disaster response, and search and rescue operations [3, 4].

Several attempts have been made to guide people who have vision and auditory impairments [5] or find themselves in situations which cause their vision and hearing to be impaired. For example, blind people use guide dogs [6] to help them find their way while fire-fighters who find themselves in low visibility conditions and encounter high auditory distractions depend on touch sensation of walls [7]. Fire-fighters have to work in low visibility conditions due to smoke or dust and high auditory distractions due to their oxygen masks and other sounds in a typical fire-fighting environment. Nowadays, they depend on touch sensation (haptic) of walls for localizing and ropes for finding the direction [7]. A personal navigation system which uses Global Positioning System (GPS) and magnetic sensors was introduced to guide blind people in [6]. The main limitation of this approach is that upon arriving at a decision making point the user has to depend on gesture based visual communication with the navigation support system, which may not be appropriate in low visibility conditions.

This paper presents identification of abstracted dynamics of haptic based human control policies and human responses on guiding/following hard reins in low visibility conditions. The extracted haptic based guidance policies can be implemented on a robot to guide a human in low visibility conditions like indoor fire-fighting, disaster response, and search and rescue operations.

A robotic guide dog with environment perception capability called Rovi has been developed [8] to guide a human with limited environment perceptions. Rovi could avoid obstacles and reach a target on a smooth indoor floor, however it encountered difficulties in uncertain environments. An auditory navigation support system for the blind is discussed in [9], where, visually impaired human subjects (blindfolded subjects) were given verbal commands by a speech synthesizer. However, speech synthesis is not appropriate for the guidance of a visually impaired person in stressful situations such as a fire emergency where background noise levels are high [10]. Ulrich *et al*. developed a guide cane without acoustic feedback in 2001 [10]. The guide cane has an ability to analyze the situation and determines appropriate direction to avoid the obstacle, and steers the wheels without requiring any conscious effort [10]. Most of the developed devices do not receive feedback from the visually impaired user. A robotic guide called MELDOG was designed by Tachi *et al*. [11] to introduce effective mobility aids for blind people. Loomis *et al*. [12] developed personal navigation system to guide blind people in familiar and unfamiliar environments. Both the MELDOG [11] and Loomis *et al*. [12] navigator could follow only commands given by the user to reach the destination. The user’s response was not taken into account for navigation. However, human response were considered for navigation in cooperative human-robot haptic navigation in [13] in unstructured environments. The method in [13] was designed for unstructured environments since the mobile robot was designed to use its on board sensors to localize in the environment and follow a path, while the blind user is tracked using an RGB-D camera placed on the mobile robot. However, our intention is to extract the guiding/following control policies which can be implemented on an intelligent agent to guide a human in unstructured environments in low visibility conditions.

Reinforcement-based learning and learning based on demonstrations are commonly used to develop control policies for human guidance by robots in low visibility environments. To identify the parameters of an auto-regressive policy we have chosen to use learning based on demonstrations. This method is safer because the controller structure and parameters can be identified offline. The identified controller can be tested for optimality and stability using simple numerical simulations, before testing online using a robotic hardware platform. In this particular scenario, the human guiders used a hard rein to guide the follower. They gave guiding signals by swinging the rein left/right in the horizontal plane, with negligible vertical movements. Therefore, the human guiding strategy can be realized by a planar 1-DOF robotic arm with a passive joint at the end point to connect the hard rein. We demonstrated the effectiveness of this idea by exporting the controller identified from human-human demonstrations directly on the planar robotic arm. The identified controller have been subjected to no modifications.

We presented haptic based human guidance in our previous studies where one person with limited auditory and visual perception of the environment is guided by an agent with full perceptual capabilities via a hard rein in [14] and [15]. In this study, we have been inspired by the guide dog scenario out of many examples of guiding via reins because in this scenario a hard rein is used to establish a connection between the dog and the visually impaired follower. To the best of our knowledge, this is the first paper showing a computational model of closed loop state dependent haptic guiding policy and state transition following policy.

We argue that any robotic assistant to a person with limited perception of the environment should account for the level of trust of the person. Trust is one of the most critical factors in urban search and rescue missions because it can impact the decisions humans make in uncertain conditions [16]. Several attempts have been made to study the level of trust of a human with limited perception of the environment [17], [18] in different environments. In a simulated game of fire-fighting, Stormont *et al*. [17] showed that the fire-fighters become increasingly dependent on robotic agents when the fire starts to spread due to randomly changing wind directions. Freedy [18] has discussed how self confidence correlates with trust of automation in human robot collaboration. Recent studies confirmed that when the trust level gets higher the activeness is increased in human robot shared control work in [19, 20]. Moreover, [20] and [16] studied how human trust can be explained quantitatively. However, our attempt is not only to quantify the human trust but also to model it in real time. In HRI, it is becoming increasingly important to consider trust of the human on the robotic counterpart in uncertain environments like real fire. In this paper we discuss a novel optimal state-dependent controller that accounts for the level in a trust of the follower as part of the state.

In summary, this paper presents the experimental methodology employed to collect data of human-human interaction via a hard rein while tracking an arbitrary path. For simplicity, hereafter “the follower” refers to the person with limited auditory and visual perception and “the guider” refers to the person with auditory and visual perception. We describe the mathematical model of the guider’s and the follower’s state dependent control policy in detail. The experimental results of human subjects along with numerical simulation results are used to show the stability of the control policy identified through experiments. The paper also discusses the virtual time varying damped inertial model to estimate the trust of the follower. Moreover, we show the validation of the extracted guiding control policy when the derived guiding policy is implemented on a planar 1-DoF robotic arm in human-robot interactions.

## Modeling

### The guider’s closed loop control policy

**Fig 1:**
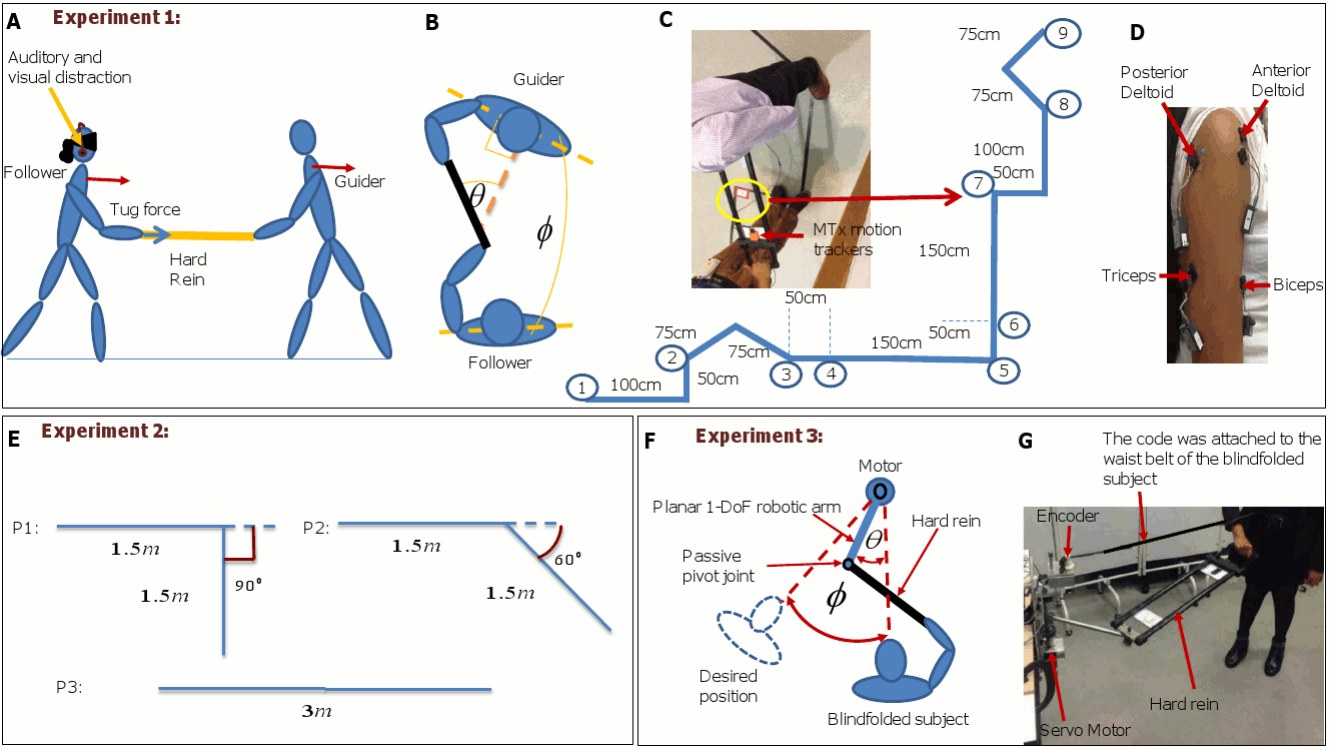
The experimental setup. Experiment 1: (A) Tracking the path by the duo: The visually and auditorily distracted follower is guided by the guider. The tug signal was given via a hard rein, (B) The state *ϕ* (the relative orientation difference between the guider and the follower) and the action *θ* (angle of the rein relative to the guiding agent), (C) The detailed diagram of labeled wiggly path on a floor, (D) EMG sensors are attached on anterior deltoid, posterior deltoid, biceps, and triceps of the subject’s arm, Experiment 2: (E) The experimental layout of trust studies: P1: Ninety degree turn, P2: Sixty degree turn, and P3: Straight path, (F) Experiment 3: *ϕ* the is relative orientation difference between the motor shaft and the guider and *θ* is the swing action in horizontal plane, and (G) Experimental setup: The hard rein was held by the human follower connected to the robotic arm across a passive joint. The cord was attached to the waist belt of the blindfolded subjects and the encoder on the shaft platform to measure the relative error (*ϕ*).

Let the state be the relative orientation between the guider and the follower given by *ϕ*, and the action be the angle of the rein relative to the sensor on the chest of the guider given by *θ* as shown in Fig 1C (see Material and methods: Experiment 1: Extracting guiding/following control policies). We model the guider’s control policy by an Auto-regressive model (AR) as a *N*-th order state dependent discrete linear controller. AR model gives us temporal (model nature) and structural (model order) relationship. The order *N* depends on the number of discrete state samples used to calculate the current action.

Then the linear discrete control policy of the guider is given by

$$\begin{array}{c} \theta_{g}\left(k\right)=\sum_{r=0}^{N-1}a_{r}^{gRe}\phi_{g}\left(k-r\right)+c^{gRe} \end{array}$$ (1)

if it is a reactive controller, and

$$\begin{array}{c} \theta_{g}\left(k\right)=\sum_{r=0}^{N-1}a_{r}^{gPre}\phi_{g}\left(k+r\right)+c^{gPre} \end{array}$$ (2)

if it is a predictive controller, where, *k* denotes the sampling step, *N* is the order of the polynomial, $a_{r}^{gRe},a_{r}^{gPre},r=1,2,\cdots ,N-1$ is the polynomial coefficient corresponding to the *r*-th state in the reactive and predictive models respectively, and *c* ^*gRe*^, *c* ^*gRe*^ are corresponding scalars.

### The follower’s state transition policy

While the guider’s control policy is represented by Eqs (1) and (2), we again model the follower’s state transition policy as an *N*-th order action dependent discrete linear controller to understand behavior of the follower. The order *N* depends on the number of past actions used to calculate the current state. Then the linear discrete control policy of the follower is given by

$$\begin{array}{c} \phi_{f}\left(k\right)=\sum_{r=0}^{N-1}a_{r}^{fRe}\theta_{f}\left(k-r\right)+c^{fRe} \end{array}$$ (3)

if it is a reactive controller, and

$$\begin{array}{c} \phi_{f}\left(k\right)=\sum_{r=0}^{N-1}a_{r}^{fPre}\theta_{f}\left(k+r\right)+c^{fPre} \end{array}$$ (4)

if it is a predictive controller, where, *k* denotes the sampling step, *N* is the order of the polynomial, $a_{r}^{fRe},a_{r}^{fPre},r=1,2,\cdots ,N-1$ is the polynomial coefficient corresponding to the *r*-th state in the reactive and predictive model respectively, and *c* ^*fRe*^, *c* ^*fPre*^ are corresponding scalars. These linear controllers in Eqs (1), (2), (3), and (4) can be regressed with the experiment 1 data obtained in the guider-follower experiments above to get the behavior of the polynomial coefficients across trials (see material and methods Experiment 1: Extracting guiding/following control policies). The behavior of these coefficients for all human subjects across the learning trials will give us useful insights as to the predictive/reactive nature, variability, and stability of the control policy learned by human guiders. Furthermore, a linear control policy given in Eqs (1), (2), (3), and (4) would make it easy to transfer the fully learned control policy to a robotic guider in low visibility conditions.

### Modeling the follower as a virtual time varying damped inertial system

In order to study how the above control policy would interact with the follower in an arbitrary path tracking task, we model the voluntary following behavior of the blindfolded human subject (follower) as a damped inertial system, where a tug force *F*(*k*) applied along the follower’s heading direction at sampling step *k* would result in a transition of position given by

$$\begin{array}{c} F\left(k\right)=M\ddot{P_{f}}\left(k\right)+\zeta \dot{P_{f}}\left(k\right) \end{array}$$ (5)

where the tug force *F*(*k*) ∈ *Re* ^2^, the virtual mass *M* ∈ *Re*, position vector *P*_*f*_(*k*) ∈ *Re* ^2^, and the virtual damping coefficient *ζ* ∈ *Re*. It should be noted that the virtual mass and damping coefficients are not those real coefficients of the follower’s stationary body, but the mass and damping coefficients felt by the guider while the duo is in voluntary movement. This dynamic equation can be approximated by a discrete state-space equation given by

$$\begin{array}{c} x(k+1)=Ax(k)+Bu(k+1) \end{array}$$ (6)

where, *k* is the sampling step, $x(k+1)=\left[\begin{array}{c} P_{f}(k+1)\\ P_{f}(k) \end{array}\right]$, $A=\left[\begin{array}{cc} \left(2M+T\zeta )/(M+T\zeta \right) & -M/(M+T\zeta )\\ 1 & 0 \end{array}\right]$, $B=\left[\begin{array}{c} T^{2}/(M+T\zeta )\\ 0 \end{array}\right]$, *u*(*k*) = *F*(*k*), and T is the sampling time.

Given the updated position of the follower *P*_*f*_(*k*), the new position of the guider *P*_*g*_(*k*) can be easily calculated by imposing the constraint ‖*P*_*f*_(*k*) − *P*_*g*_(*k*)‖ = *L*, where *L* is the length of the hard rein. We obtain the guider’s location assuming that the guider is always on the known desired path. Therefore, given a follower’s position *P*_*f*_(*k*) the intersection of the desired path and the circle with center at *P*_*f*_(*k*) and radius *L* will give the guider’s location.

## Results

### Experiment 1: Extracting guiding/following control policies

We conducted Experiment 1 with 15 naive pairs to understand how the coefficients of the control policy relate to states *ϕ* and actions *θ* given in Eqs (1), (2), (3), and (4) settle down across learning trials. In order to have a deeper insight into how the coefficients in the discrete linear controller in Eqs (1), (2), (3), and (4) change across learning trials, we ask whether 1) the guider and the follower tend to learn a predictive/reactive controller across trials, 2) the order of the control policy of the guider in Eqs (1) and (2) and the order of the control policy of the follower in Eqs (3) and (4) change over trials, and if so, what its steady state order would be.

#### Adoption of wave families for action and state vector profiles

**Fig 2:**
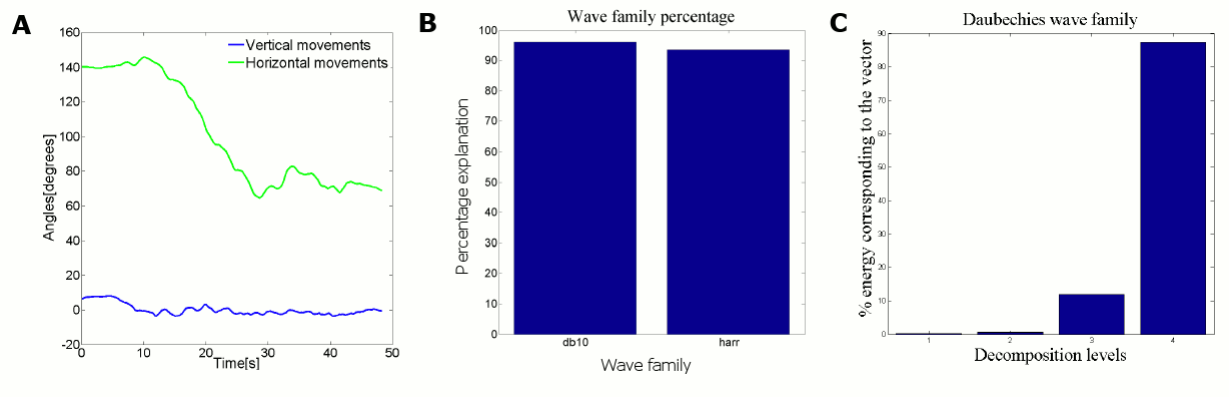
Selection of wavelet family for guiding agent action and state vectors. (A) The vertical movements and horizontal action vector for the guider in a representative trial, and (B) The percentage of energy representation of action vector of all subjects in all trials for db10 and harr wavelet families, and (C), The percentage of energy corresponding to 1 to 4 decomposition levels in db10 wave family. The averaged action vector across the all subjects over trials are taken.

Since the raw motion data have noise, we used Wavelet Toolbox (The MathWorks Inc.) to reduce the noise in the action and the state vectors to find regression coefficients. The guider’s action is a continuous swing and pull on the horizontal plane. For clarity, we plotted the guider’s arm movement in horizontal and vertical planes for a random trial as shown in Fig 2A. The vertical movements are very too slow compared to horizontal movements. Therefore, we consider only horizontal movements to represent the arm action. We chose the Daubechies wave family (for sinusoidal waves) [21] in the wavelet analysis. According to previous studies [22, 23] human arm movements are continuous and smooth. Therefore, a continuous mother wavelet (*db10*) is taken to represent the swing actions in wavelet analysis. For further clarity, we compared the percentage of energy representation of *db10* and *harr* wave families as shown in Fig 2B. Considering higher percentage value, we selected *db10* for our swing type action analysis.

Then different decompression levels were tested for *db10*. The percentage of energy corresponding to approximation for different decompression levels was found to be 99.66%, 93.47%, and 86.73% for decompression levels 4, 8, and 15 respectively. The highest percentage of energy was gained when the decompression level is 4.

Fig 2C shows the percentage energy corresponding to decomposition levels 1–4 of the action vector. We use the 4^th^ decomposition level for action vector analysis since this level has the highest percentage value (88%). The same procedure was tested for state vector profile and the results correspond to those obtained for the action vector profile. Based on the results we adopt 4^th^ decomposition level of *db10* wave family to analyze raw data of the action and the state.

#### Determination of the guider’s control policy

Hereafter, the 4^th^ decomposition level of *db10* of action *θ* and state *ϕ* vectors are used for regression in Eqs (1) and (2). Once the coefficients of the polynomial in Eqs (1) and (2) are estimated, the best control policy (Eqs (1) or (2)), and the corresponding best order of the polynomial should give the best *R* ^2^ value for a given trial across all subjects. Here, twenty experimental trials were binned to five for clarity.

#### Determination of predictive/reactive nature of the guider’s control policy

**Fig 3:**
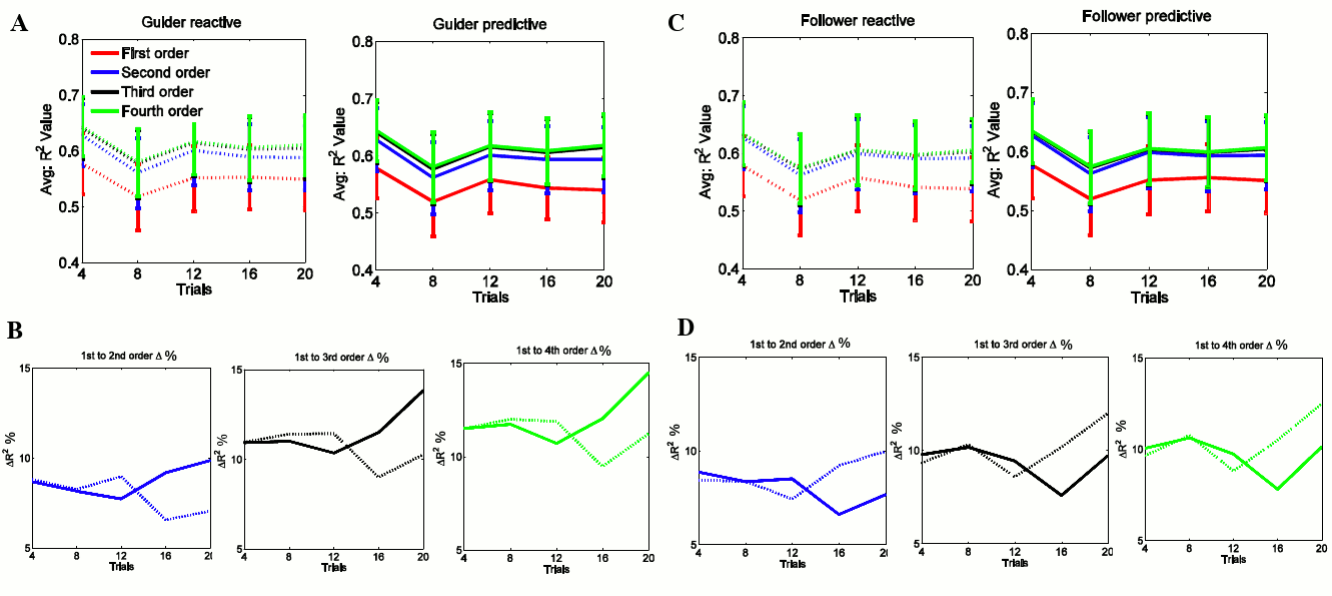
*R* ^2^ values from 1^st^ order to 4^th^ order polynomials for the guider and the follower. reactive models (dashed line) and predictive models (solid line): (A) and (C) are the *R* ^2^ value variation of the reactive and predictive from 1^st^ to 4^th^ order polynomials over trials for the guider and the follower respectively. (B) and (D) are the percentage (%) differences of *R* ^2^ values of 2^nd^ to 4^th^ order polynomials with respect to 1st order polynomial for the guider’s and the follower’s control policies respectively: 1st to 2^nd^ order (blue), 1st to 3^rd^ order (black), 1^st^ to 4^th^ order (green).

Coefficients of Eqs (1) and (2) were estimated from 1^st^ order to 4^th^ order polynomials in Fig 3A to select best fit policies. Dashed line and solid line were used to denote reactive and predictive models respectively. From Fig 3A, we can observe that the R^2^ values corresponding to the 1^st^ order model in both Eqs (1) and (2) are the lowest. The relatively high R^2^ values of the higher order models suggest that the control policy is of order > 1. Therefore, we consider the percentage (%) differences of *R* ^2^ values of higher order polynomials relative to the 1^st^ order polynomial for both Eqs (1) and (2) to assess the fitness of the predictive control policy given in Eq (2) relative to the reactive policy given in Eq (1). Fig 3B shows that the marginal percentage (%) gain in *R* ^2^ value (%△*R* ^2^) of 2^nd^, 3^rd^, and 4^th^ order polynomials in Eq (2) predictive control policy (solid line) grows compared to those of the reactive control (dashed line) policy in Eq (1). Therefore, our conclusion is that the guider gradually prefers to employ a predictive control policy than on a predictive control policy than a reactive one.

#### Determination of the model order of the guider’s control policy

The data is not sufficient to test whether the population follows normal distribution after binning. The Mann-Whitney test does not require the assumption that the differences between the two samples are normally distributed. Therefore, the non-parametric Mann-Whitney U test (*α* = 0.05) was conducted to test significance. The percentage (%) gain of 3^rd^ order polynomial is highest compared to 2^nd^ and 4^th^ order polynomials as shown in Table 1 by numerical values and Fig 3B. There is a statistically significant improvement from 2^nd^ to 3^rd^ order models (*p* = 0.008), while there is no significant information gain from 3^rd^ to 4^th^ order models (*p* = 0.54). Means that the guider predictive control policy is more explained when the order is *N* = 3. No more information is added for higher orders after *N* = 3. Therefore, hereafter, we use 3^rd^ order predictive control policy to explain the guider’s control policy.

**Table 1:** Guider predictive △*R* ^2^% of 2^nd^ to 4^th^ order polynomials w.r.t 1^st^ order. Statistical significance was computed using the Mann-Whitney U test (*α* = 0.05).

Trial No:	2^nd^ order	3^rd^ order	4^th^ order	*p* values
4	8.94	11.37	11.97	
8	8.26	10.98	11.62	
12	7.81	10.36	10.74	*p*(2^*nd*^ ↔ 3^*rd*^) < 0.008*,
16	9.38	11.68	12.25	*p*(3^*rd*^ ↔ 4^*th*^) > 0.5
20	9.74	14.00	14.70	

Therefore the guider’s control policy can be written

$$\begin{array}{c} \theta_{g}\left(k\right)=a_{0}^{gPre}\phi_{g}\left(k\right)+a_{1}^{gPre}\phi_{g}\left(k+1\right)+a_{2}^{gPre}\phi_{g}\left(k+2\right)+c^{gPre} \end{array}$$ (7)

#### Determination of the follower’s state transition policy

Next we try to understand the follower’s state transition policy in response to guider’s actions, hereafter referred to as follower’s state transition policy.

#### Determination of predictive/reactive nature of the follower’s state transition policy

We used experimental data for state *θ* and action *ϕ* in Eqs (3) and (4) to extract features of the follower’s state transition policy from 1^st^ to 4^th^ order polynomials over trials as shown in Fig 3C. Here, we used the same mathematical and statistical method as in the guider’s model. Fig 3C shows that the marginal percentage (%) gain in R^2^ value (%△*R* ^2^) of 2^nd^, 3^rd^, and 4^th^ order polynomials in Eq (3) reactive control policy (dashed line) grows compared to that of the predictive control policy (solid line) in Eq (4). Therefore, our conclusion is that the follower gradually more employs on a reactive policy than a predictive one.

#### Determination of the model order of the follower’s state transition policy

The percentage (%) gain of 2^nd^ order polynomial is highest compared to 3^rd^ and 4^th^ order polynomials as shown in Table 2 by numerical values. Interestingly, there is no statistically significant improvement from 2^nd^ to 3^rd^ order models (*p* = 0.42) or from 3^rd^ to 4^th^ order models (*p* = 0.54). Therefore, we can say the follower reactive policy is more explained when the order is *N* = 2. Therefore, hereafter, we consider 2^nd^ order reactive policy to explain follower’s state transition policy.

**Table 2:** Follower reactive △*R* ^2^% of 2^nd^ to 4^th^ order polynomials w.r.t 1^st^ order. Statistical significance was computed using the Mann-Whitney U test (*α* = 0.05).

Trial No:	2^*nd*^ order	3^*rd*^ order	4^*th*^ order	*p* values
4	8.58	9.57	9.91	
8	8.31	10.33	10.77	
12	7.41	8.46	8.70	*p*(2^*nd*^ ↔ 3^*rd*^) > 0.1,
16	9.45	10.21	10.51	*p*(3^*rd*^ ↔ 4^*th*^) > 0.5
20	9.96	11.82	12.29	

The follower’s state transition policy can be written as,

$$\begin{array}{c} \phi_{f}\left(k\right)=a_{0}^{fRe}\theta_{f}\left(k\right)+a_{1}^{fRe}\theta_{f}\left(k-1\right)+c^{fRe} \end{array}$$ (8)

#### Polynomial parameters of auto-regressive state dependent behavioral policies of the duo

**Fig 4:**
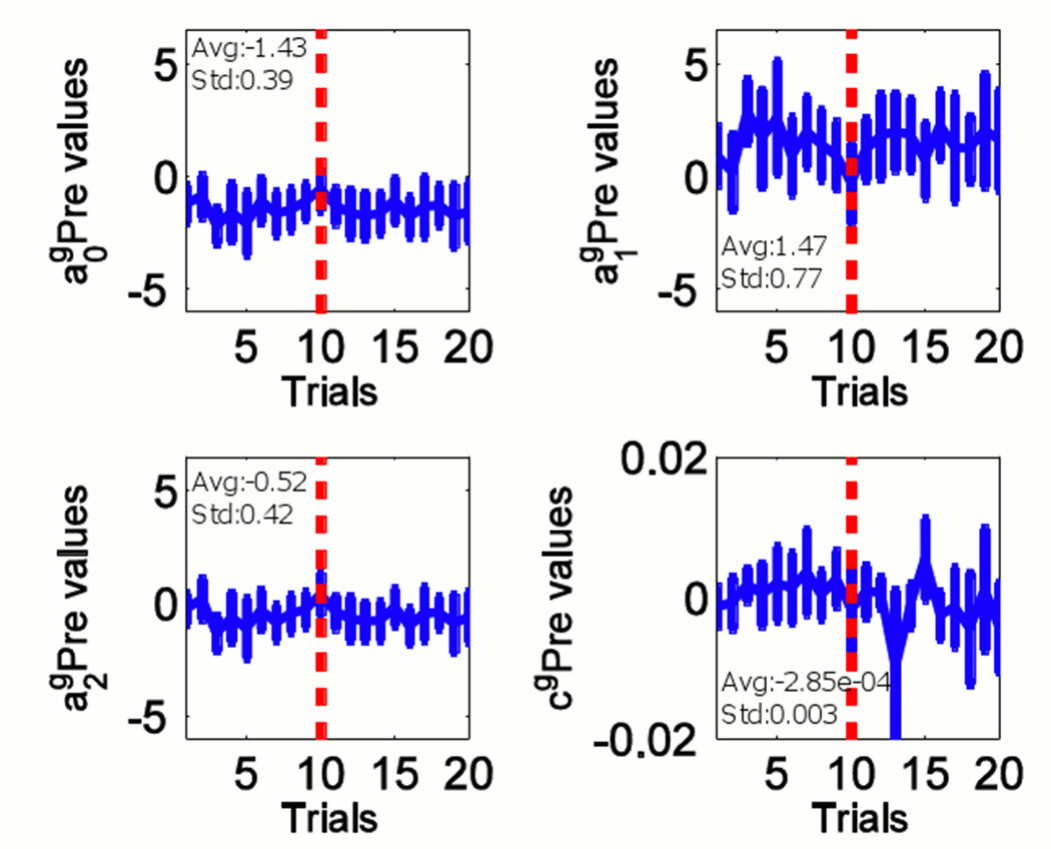
The representation of coefficients of the 3^rd^ order auto regressive predictive controller of the guider. 10^th^ trial is marked by a red dashed line. Trials from 10^th^ to 20^th^ were only taken for the simulation in Fig 10.

**Fig 5:**
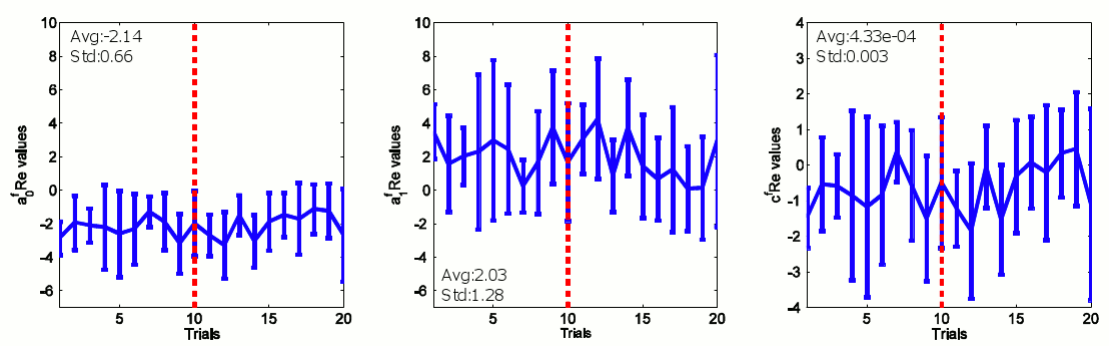
The evolution of coefficients of the 2^nd^ order auto regressive reactive controller of the follower.

We proceed to explore how the polynomial parameters of the guider’s 3^rd^ order predictive and the follower’s 2^nd^ order reactive policies can change across learning trials in Eqs (2) and (3) for the guider and the follower respectively. We notice in Figs 4 and 5 that the history of the polynomial coefficients fluctuates within bounds for both the guider predictive and the follower reactive. The average and standard deviation values of the coefficients are labeled in Figs 4 and 5 (denoted by avg: and std: respectively). This may be the result of the variability across subjects as well as of the variability of parameters across trials. Therefore, we could estimate the above control policy as a bounded stochastic decision making process.

#### Optimality of muscle recruitment

**Fig 6:**
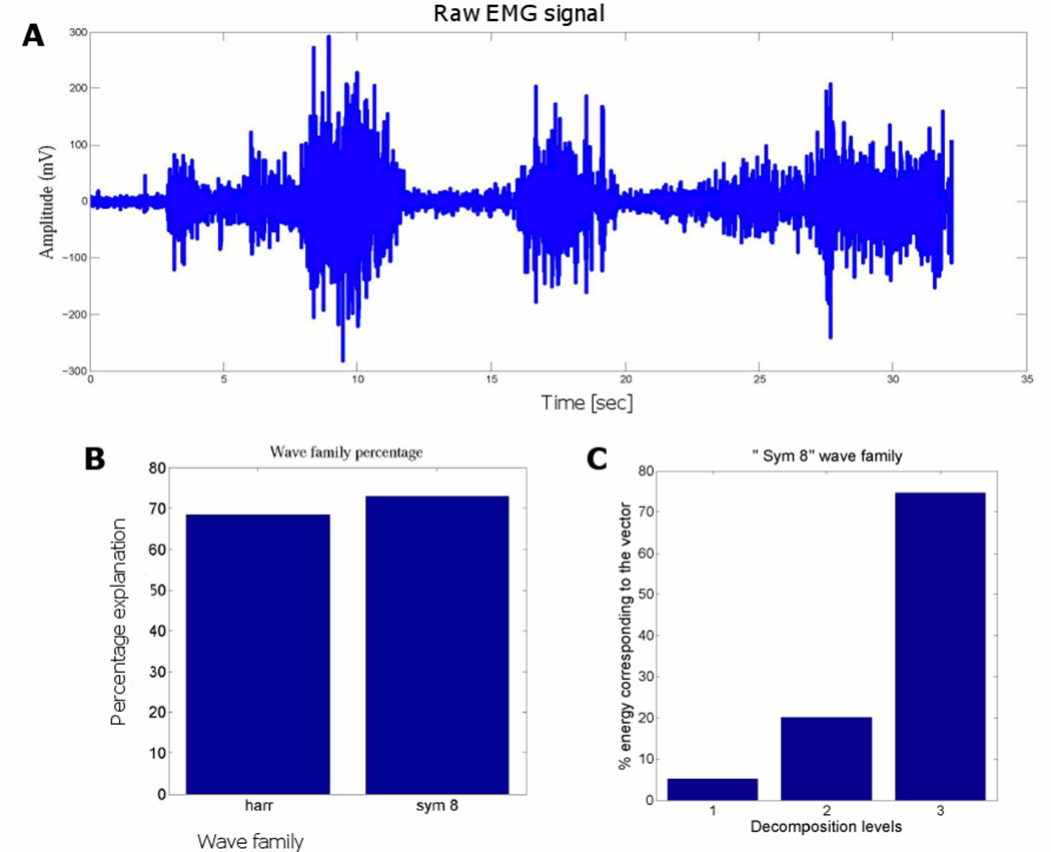
Selection of wavelet family for EMG vector. (A) A representative raw EMG signal from the guider, (B) The percentage of energy representation for harr and sym8 wavelet families for raw EMG signal in Fig 7A, (C) The percentage of energy corresponding to 1 to 3 decomposition levels for sym8 wave family for the EMG signal in Fig 7A.

To understand the optimality of muscle activation, we proceed to study the responsibility assignment of muscles from EMG recordings. We used the Wavelet Toolbox to reduce the noise of the raw EMG data (The MathWorks Inc.). The raw EMG signal is a sinusoidal continuous wave as shown in Fig 6A. Therefore we chose the *sym8* in Symlets wave family (The MathWorks Inc) [21] for EMG analysis. The percentage of energy corresponding to *sym8* (Symlets) and *harr* (Harr) is 72.9% and 68% respectively. This is demonstrated in a bar chart shown in Fig 6B. Considering the highest energy percentage, we selected *sym8* for our EMG wave analysis. Then different decompression levels were tested for *sym8*. The percentages of energy corresponding to approximation for different decompression levels were found to be 99.52%, 95.97%, 92.05%, 85.41%, and 20.36% for decompression levels 3, 4, 5, 6 and 7 respectively. The highest percentage of energy was gained when the decompression level is 3. Fig 6C shows the percentage energy corresponding to the 1 to 3 decomposition levels of the EMG signal. Since the 3^rd^ decomposition level has the highest percentage of energy, we use it hereafter to analyze raw EMG data.

#### Behavior of antagonist muscles

**Fig 7:**
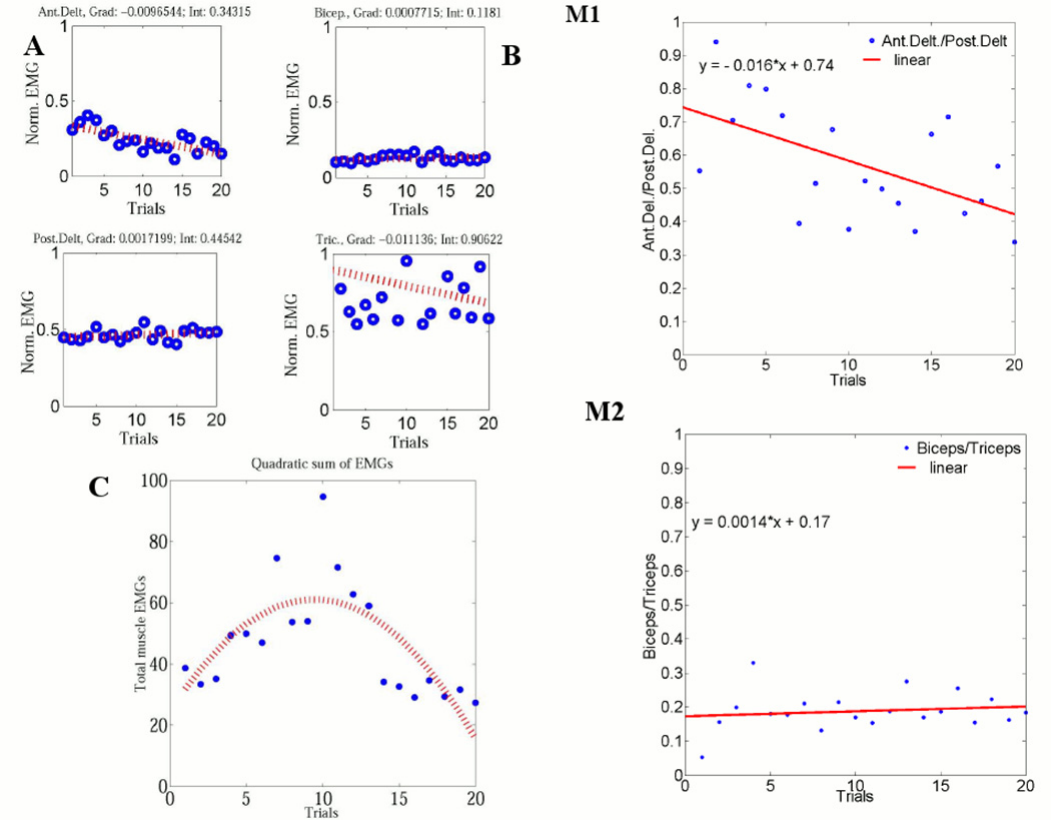
The behavior of the average normalized muscle EMGs. (A) Average normalized muscle EMG anterior deltoid, posterior deltoids, biceps, and triceps. The gradient and intercept of individual muscles are (−0.005, 0.315), (0.004,0.426), (0.001, 0.133), and (−0.013, 0.995) for anterior deltoid, posterior deltoid, biceps, and triceps respectively, (B) Frontal and dorsal muscle ratio: M1- biceps triceps muscle ratio, M2- anterior deltoid posterior deltoid muscle ratio, and (C) The behavior of this cost indicator *J* of the 2^nd^ order best fit curve for average EMGs of all four muscles of the ten subjects across trials.

When the guider takes the arm action in horizontal plane as shown in Fig 2A, it can be pushing/ pulling or swinging in horizontal plane. The anterior deltoid and posterior deltoid are recruited to perform pushing and pulling actions. The guider can use the elbow joint in two different ways: one is to swing the rein in the vertical plane by flexing the elbow without moving the shoulder joint, and the other is to pull the rein if the elbow is flexed in synchrony with a shoulder joint flexion. To understand the muscle recruitment, we plotted the average normalized activation of each individual muscle and the averaged normalized EMG ratio between frontal and dorsal muscles in all trials as shown in Fig 7A and Fig 7B respectively. There is a downward trend in the ratio of anterior deltoid and posterior deltoid muscles in all trials (Fig 7B:M1), while there is an upward trend in the ratio of biceps and triceps muscles has a upward trend in all trials (Fig 7B:M2). This could indicate that a forward model of task dynamics is learnt across trials. Kolmogorov-Smirnov test proved the average muscle distribution comes from a normal distribution. Therefore significance was tested by t-test. Here, the significance test was conducted between the first 5 trials and last 5 trials of M1 and M2 using single tailed t-test. The results show that the ratio of first five trials and last five trials of anterior deltoid and posterior deltoid (M1) are significantly different (*p* = 0.00004) while there is no significance between the ratio of first five trials and last five trials of biceps and triceps (M2) (*p* = 0.85). This suggests that the forward model [24, 25] that predicts the consequence of guiding actions accounts for the activity of deltoids than the elbow joint. This may be due to the fact that the elbow joints is mainly responsible to keep the guider’s actions on the horizontal plane.

#### Behavior of total EMG over trials

To obtain an estimation of the total energy consumed during guiding, we compute the average EMG for all four muscles of all fifteen pairs that reflects the average energy consumed in a trial given by $J=\sqrt{\sum_{i=1}^{4}\sum_{j=1}^{S_{N}}EMG_{ij}^{2}}$, where *S*_*N*_ is the number of subjects, *EMG*_*ij*_ is the average rectified EMG of the *i*th muscle of the *j*th subject. The behavior of this energy consumption indicator *J* is shown in Fig 7C. We can clearly observe from the 2^nd^ order best fit curve that *J* increases to a maximum in the first half of the trials and decreases in the last 10 trials. This suggests that optimization is a non-monotonic process. During the first half of the trials, it may have given priority to predictive control policy order selection (Eq (7)) and the formation of the forward model to predict follower’s state into the future than to optimization in the muscle activation space, which is also reflected in the behavior of *R* ^2^ values in Fig 3. Once the optimal order is selected, subjects exhibit monotonic optimization in the muscle activation space as seen in the last 10 trials of Fig 7C, with a corresponding increase of *R* ^2^ values in Fig 3.

### Experiment 2: Modeling the follower’s trust in different paths

Here our intention is to incorporate the instantaneous trust level of the follower in the state-space of the closed loop controller. We show the results of 14 naive subjects’ variability of voluntary movements towards a blindfolded follower in a virtual damped inertial dynamic system. Our attempt is to address the question of how the follower’s trust towards the guider should be accounted for in designing a closed loop controller. Here, we argue that the trust of the follower in any given context should be reflected on how compliant his/her voluntary movements are to the instructions of the guider.

**Fig 8:**
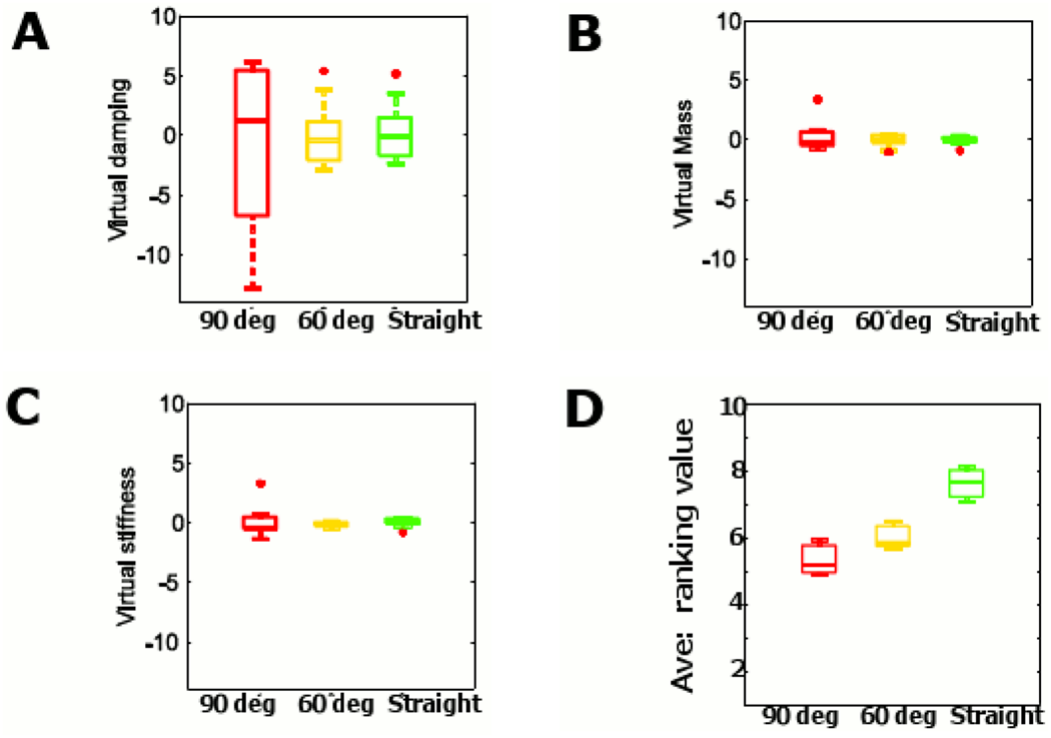
Regression coefficients in Eq (9) of different paths. (A) Virtual damping coefficient for paths: 90° turn (red), 60° (yellow) turn, and straight path (green). The average values are 3.055, 1.605, and −0.586 for 90° turn, 60° turn. and straight path respectively, (B) Virtual mass coefficient for paths: 90° turn (red), 60° turn (yellow), and straight path (green). The average values are 2.066, −0.083, and 0.002 for 90° turn, 60° turn, and straight path respectively, (C) Virtual stiffness coefficient for paths: 90° turn (red), 60° (yellow) turn, and straight path (green). The average values are 0.0325, −0.1385, and 0.0117 for 90° turn, 60° turn. and straight path respectively, and (D) The follower’s response towards the trust scale: The trust scale varies from 1 to 10 from the lowest to the highest.

The experimental results of 14 pairs of subjects in three types of paths—90° turn, 60° turn, and straight—are shown in Fig 8. Here we extracted motion data within a window of 10 seconds around the 90° and 60° turns, and for fairness of comparison, we took the same window for the straight path for our regression analysis to observe the virtual damping coefficient, virtual stiffness coefficient and the virtual mass in three different paths. Fig 8A shows the variability of the virtual damping coefficient, and Fig 8B shows the virtual mass for the above three contexts. We can notice from Fig 8A that the variability of the virtual damping coefficient is highest in the path with a 90° turn while there is less variability in the 60° turn path and least variability in the straight path.

When the follower voluntarily moves forward according to the small tug-signal of the guider, any increase of force felt by the guider must come from a reduction in the voluntary nature of followers movement. Therefore we modeled the follower as a virtual damped inertial model. To represent the variable voluntary nature of the follower, we did not consider virtual stiffness because the original location is irrelevant in a voluntary movement. However, we tested the variability of virtual stiffness adding the stiffness to Eq (5).

Then the Eq (5) becomes

$$\begin{array}{c} F\left(k\right)=M\ddot{P_{f}}\left(k\right)+\zeta \dot{P_{f}}\left(k\right)+kP_{f}\left(k\right) \end{array}$$ (9)

Fig 8C shows the variability of the virtual stiffness for 90° turn, 60° turn, and straight path. The variability of the stiffness and the mass are low as shown in Fig 8B and Fig 8C while variability of damping coefficient is high as shown in Fig 8A. In Fig 8A, in Fig 8B, and in Fig 8C the average values of the virtual damping coefficient, the virtual mass, and the virtual stiffness distribution in straight path are lowest. This shows that the trust level of the follower is greater in the straight path. Table 3, Table 4, and Table 5 show the results of Mann-Whitney U test for different paths (90° turn, 60° turn, straight path) of coefficients in Eq (9). Results in Table 3 show that the virtual damping coefficient in 90° turn was significantly different from that in straight path (*p* = 0.009). Moreover, virtual damping coefficient in 60° turn was also significantly different from that in straight path (*p* = 0.01). There was no statistically significant difference between the virtual damping coefficient with the 90° and 60° turns (*p* = 0.90). The virtual mass distribution in Eq (9) is shown in Fig 8B. Significance test results show that the straight path is different from the path with the 90° turn (*p* = 0.006). However, no significant differences between the 60° turn path and the straight path (*p* = 0.8). This may be due to the fact that the follower shows more trust in following the guider down a straight path than in paths with turns.

**Table 3:** Virtual damping coefficients. Statistical significance was computed using the Mann-Whitney U test (*α* = 0.05).

Paths	Mean	
90° turn	3.055	*p*(90° *turn* ↔ 60° *turn*) > 0.6,
60° turn	1.605	*p*(60° *turn* ↔ *Straightpath*) < 0.02*,
Straight path	−0.586	*p*(90° *turn* ↔ *Straightpath*) < 0.01*

**Table 4:** Virtual mass coefficients. Statistical significance was computed using the Mann-Whitney U test (*α* = 0.05).

Paths	Mean	
90° turn	2.066	*p*(90° *turn* ↔ 60° *turn*) > 0.8,
60° turn	−0.083	*p*(60° *turn* ↔ *Straightpath*) > 0.7,
Straight path	0.002	*p*(90° *turn* ↔ *Straightpath*) < 0.01*

**Table 5:** Virtual stiffness coefficients. Statistical significance was computed using the Mann-Whitney U test (*α* = 0.05).

Paths	Mean	
90° turn	0.0325	*p*(90° *turn* ↔ 60° *turn*) < 0.05*,
60° turn	−0.1385	*p*(60° *turn* ↔ *Straightpath*) < 0.05*,
Straight path	0.0117	*p*(90° *turn* ↔ *Straightpath*) < 0.05*

However, the virtual stiffness is significantly different in the 90° turn path compared to the straight path (*p* = 0.002) and 60° turn (*p* = 0.004). Moreover, 60° turn is significantly different from straight path (*p* = 0.001). Even though the virtual stiffness is significantly different for three defined paths the variability is very low. However, the variability of virtual damping coefficient is higher than virtual mass and stiffness. Therefore, these results confirm that the follower’s trust level is reflected in the time varying parameter of the virtual damped inertial system. We also note that the virtual damping coefficient reflects more accurately the level of trust than the virtual mass or stiffness.

We conclude that the virtual damping coefficient can be a good indicator to control the push/pull behavior of an intelligent guider using a feedback controller of the form given in Eq (10), where F(k) is the pushing/pulling tug force along the rein from the human guider at *k* ^th^ sampling step, M is the time varying virtual mass, *M*_0_ is its desired value, *ζ* is the time varying virtual damping coefficient, *ζ*_0_ is its desired value, and k is the sampling step.

$$\begin{array}{c} F\left(k+1\right)=F\left(k\right)-\left(M-M_{0}\right)\ddot{P_{f}}\left(k\right)-\left(\zeta -\zeta_{0}\right)\dot{P_{f}}\left(k\right) \end{array}$$ (10)

Human subjects consistently confirmed that their trust level in following the guider dropped as they moved from the straight path, to the 60° turn path and further decreased when they took the 90° turn path. Moreover, we present the followers’ response towards the defined trust scale (see material and methods) in Fig 8D, where the average trust scale values across all the subjects for straight, 60° turn, and 90° turn are shown in Fig 8D. The variability of 90° turn is higher than that of the 60° turn and straight paths as shown in Fig 8D. For further clarity, the significance was computed by Mann-Whitney U test as shown in Fig 8D. The results show that between straight and 90° turn (*p* = 0.01) and straight and 60° turn are significantly different (*p* = 0.03). The followers response after each trial confirms that the follower shows more confidence when following the guider along a straight path than along paths with 90° and 60° turns.

#### Developing a closed loop path tracking controller incorporating the follower’s trust level

We combine the guider’s 3^rd^ order predictive policy in Eq (7) to control the swing movement of the hard rein with the tug force modulation rule in Eq (10) to form a complete controller that accounts for the state of the follower that indicating his/her trust level.

We use the last 10 trial’s coefficients values (marked on Figs 4 and 5 by red dashed line) to calculate the statistical features of the regression coefficients in order to make sure the model reflects the behavior of the human subjects at a mature learning stage. In this stage, we assume that the distribution of the coefficients as a normally distributed random variable. Therefore, the model parameters were then found to be: *a*_0_ = *N*(−1.6784, 0.1930^2^), *a*_1_ = *N*(1.4710,0.5052^2^), *a*_2_ = *N*(−0.5295,0.5052^2^), and *c* = *N*(−0.4446,0.2643^2^).

In order to ascertain whether the control policy obtained by this system identification process is stable for an arbitrarily different scenario, we conducted numerical simulation studies forming a closed loop dynamic control system of the guider and the follower using the control policy given in Eq (7) together with the discrete state space equation of the follower dynamics given in Eq (6). The length of the hard rein *L* = 0.7*m*, the mass of the follower *M* = 10*kg* with the damping coefficient *ζ* = 4*Nsec*/*m*, the magnitude of the force exerted along the rein was 5N, and the sampling step *T* = 0.2.

**Fig 9:**
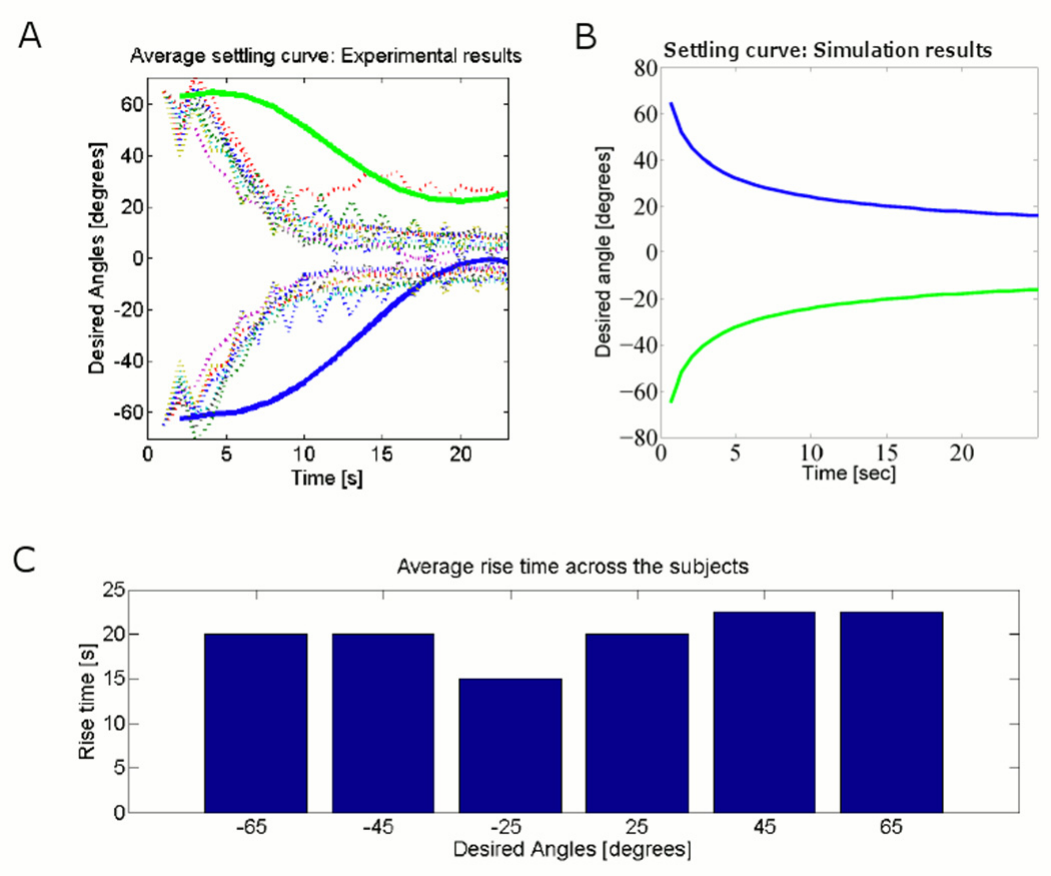
Experimental setup and results to validate the guider’s control policy. (A). The experimental results of completion the task for 10 naive subjects for the desired angles +65° and −65°. The individual subjects completion are shown by dashed lines. The average task completion fitted curves across all subjects are shown by a solid line, (B) Simulation results for the task completion for the desired angles +65° and −65°, and (C) Average rise time across 10 subjects for desired angles +65°, +45°, +25°, −65°, −45°, and −25°.

Moreover, when the follower’s desired angles are *ϕ*(0) = +65° and *ϕ*(0) = −65°, we notice that the simulated damping behavior of followers error reduction from Fig 9B. This confirms that the guiding control policy is comparable with the human-robot experimental results in Fig 9A with simulation results in human demonstration experiments in Fig 9B for reaching same desired angles +65° and −65°.

**Fig 10:**
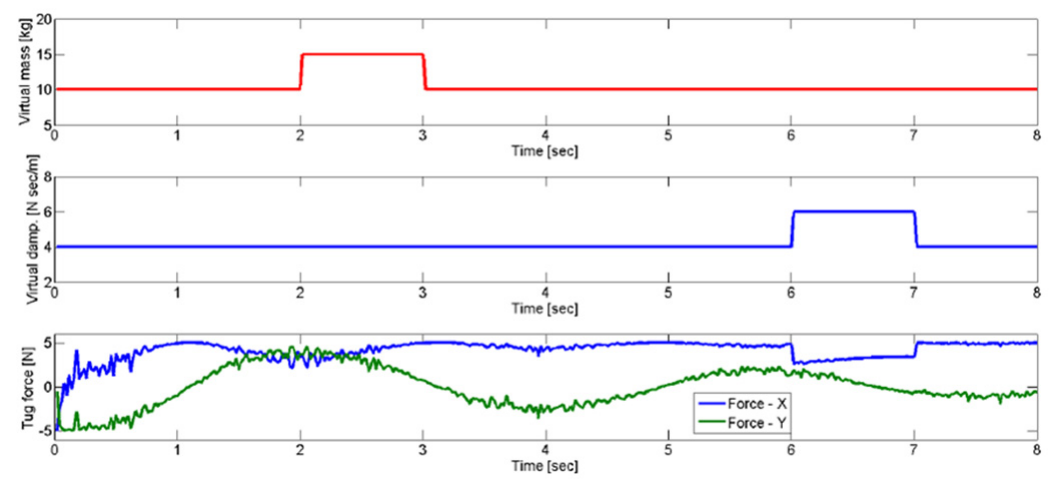
Simulation results. The tug force variation of the follower in order to achieve a sudden change of the virtual mass *M* = 15[kg] from *t* = 2s to *t* = 3s and the virtual damping coefficient *ζ* = 6[Nsec/m] from *t* = 6s to *t* = 7s. *F*_*X*_ and *F*_*Y*_ are forces in X and Y directions.

To understand the variability of the virtual model parameters based on the model, we set the virtual mass *M* = 15*kg* from *t* = 2*sec* to *t* = 3*sec* and the virtual damping coefficient *ζ* = 6*Nsec*/*m* from *t* = 6*sec* to *t* = 7*sec* to observe tug force variation in Eq (6) as shown in Fig 10. The tug force variation in Fig 10 shows that the virtual damping coefficient more influenced to vary the tug force than the virtual mass. The results suggest that the virtual model parameter can be used to demonstrate the level of trust of the follower.

### Experiment 3: Validating the guider’s control policy

#### The guider’s closed loop control policy validation

We implemented the guider’s control policy in Eq (7) to generate a tug force from the planar 1-D of freedom robotic arm in order to guide the blindfolded follower as shown in Fig 1G. The experimental results of the trials involving 10 naive subjects show that the closed loop controller minimizes the error in regards to guiding the subject to the desired target as shown in Fig 9A. The individual subject’s task completion is represented by a dashed line while the average fitted settling curve across all subjects is represented by a solid line in Fig 9A. Moreover, a comparison of experimental results (Fig 9A) and simulation results (Fig 9B) suggest that the open loop guiding controller can minimize the following error to bring the human subject in to the desired point. There is no significant difference between the average distribution of experimental recording (*p* = 0.857) in Fig 9B across the subjects and the simulation (*p* = 0.067) in Fig 9B for reaching −65° and +65°. The significance test results confirm the validation of the proposed virtual damped inertial model given in Eq (10) for the follower. Combining the trust studies in Fig 8A and Fig 8B, and the simulation in Fig 9A and Fig 9B, we conclude that the virtual damping coefficient would be used as an indicator to represent the human follower’s trust level.

We show how the average error *ϕ* was reduced over time across the trials for ten subjects for desired angles −65° and +65° as shown in Fig 9A and Fig 9B for experimental data and simulation respectively. The results show that the implemented guider’s control policy is able to bring the blindfolded subject into the desired position and settle down in a reasonable time. For clarity, we demonstrate the average rise time across all subjects for the given six desired angles as shown in Fig 9C. The desired angles are −65°, −45°, −25°, +25°, +45°, and +65°. We consider the rise time measured for the number of commands to reach from 10% to 90% of the desired angles. We use stepinfo function (MATLAB 2012b) to extract the rise time. A single trial was run for 90 seconds. The experimental results show that within reasonable time the subjects can reach to the desired angle and settle down. This again confirms that implemented controller can bring subjects into the desired positions and settle down in a reasonable time.

## Discussion

This study was conducted to explore how two human participants interact with each other using haptic signals through a hard rein to achieve a path tracking goal when one partner (the follower) is blindfolded, while the other (the guider) receives full state feedback of the follower.

### The duo’s policies

If an intelligent agent (man/machine) is given the task to guide such a follower using only a hard rein, the guiding agent should learn a control policy that can effectively manage the variability of follower’s behavior [26]. In this study, we conducted experiments to understand how two human subjects interact with each other using haptic signals through a hard rein to achieve a path tracking goal when one partner was cut off from auditory and visual feedback from the environment (the follower), while the other person with environmental perception (the guider) gets full state feedback of the follower to find variability of movement and uncertainty of the behavior.

The *R* ^2^ values of the guider’s predictive and the follower’s reactive behavioral policies increased over the course of the trials, as shown in Fig 3A and Fig 3C. The significance test results among different orders of auto-regressive policies confirm that the guider’s policy is best approximated by a 3^rd^ order model while the follower’s state transition policy is best approximated by a 2^nd^ order model. The results suggest that in general, the guider depends on more information than the follower. The follower’s 2^nd^ order reactive and the guider’s 3^rd^ predictive control policies suggest that a reactive behavior does not need as many past states as in a predictive behavior to take actions. The proposed control policy based on human-human demonstrations is mainly intended for use in robots which guide people with good vision working in low visibility environments as in fire-fighting and other disaster response operations.

Variability is an indispensable feature in human behavior [27]. Therefore, we set out to understand the specific properties of variability of human guiding behavior in this particular task by observing the variation of polynomial coefficients in Eqs (2) and (3) across trials. By modeling the control policy learned by the guiding agent as a discrete state dependent auto-regressive function, we found that guiding agent learns a stochastic stable control policy across 20 trials as shown in Figs 4 and 5. These results are consistent with those of previous studies on stochastic human behavior [27–29] in similar contexts.

### The human follower’s trust

Previous studies on human trust on a guiding agent have shown that humans tend to depend entirely on the guiding agent when they are in hazardous environments [17] until a sudden change occurs [30]. This implies that the degree of compliance in a follower should diminish if the follower loses trust in the guiding agent. By modeling the impedance of the follower as a virtual inertial damped stiffness system, we then considered the variability of the follower’s impedance parameters (the virtual mass, damping, and stiffness coefficients) at different turn angles. Regarding the three types of paths shown in Fig 8 the blindfolded subjects who played the role of the follower confirmed that their trust in following the guider was highest in the straight path while it decreased in the other paths with the lowest trust level recorded in the path with the 90° turn. The results of virtual impedance parameters in Eq (9) are shown in Fig 8A, Fig 8B, and Fig 8C. Our experimental results of human subjects also show that the variability of the virtual damping coefficients correlates more with the complexity of the path in Fig 8—reflecting the trust level of the follower—than that of the virtual mass or stiffness coefficients. Therefore, we consider the follower as virtual damped inertial system as in Eq (5). Fig 8A, Fig 8B, and Fig 8C show that the higher trust of the follower in the straight path results in a lower average value of the virtual damping coefficient. When the follower’s trust decreases during the 90° and 60° turns, the guider has to exert a higher tug force to lead the follower to the desired trajectory. This results to higher average values for the virtual mass, virtual damping coefficient, and virtual stiffness coefficients.

Moreover, the experimental average trust scale test results in Fig 8C suggest that the follower’s trust decreases in 90° and 60° turns.

Once the parameters of the Eq (2) are known, the damped inertial model of the voluntary movement of the follower can be combined to form a complete state dependent controller that accounts for the trust level of the follower as given by $\left[\begin{array}{c} F(k+1)\\ \theta (k+1)) \end{array}\right]=\left[\begin{array}{cc} 1 & 0\\ 0 & 1 \end{array}\right]\left[\begin{array}{c} F(k))\\ \theta (k) \end{array}\right]+\left[\begin{array}{c} \left(M-M_{0}){\overset{..}{P}}_{f}(k)-(\zeta -\zeta_{0}){\dot{P}}_{f}(k)\right)\\ \sum_{r=0}^{N-1}a_{r}^{Pre}\phi (k+r)+C^{Pre} \end{array}\right]$ where *M*_0_ and *ζ*_0_ are desired mass and desired damping coefficients respectively. This complete state dependent controller can be readily implemented in a potential human-robot interaction scenario.

Therefore, our results from human-human demonstrations provide useful design guidelines to human-robot interaction that should account for the real-time trust level of the human counterpart. In a human-robot interaction scenario, such as one involving a fire-fighter being guided by a robot through thick smoke, the estimate of the followers’ trust using the above method could be used to change acceleration/deceleration of the intelligent agent.

### Arm muscle recruitment for cost minimization

Previous work [31] has proved that the total muscle activation for a single task decreased over learning trials [31]. From the 2^nd^ order best fit curve for the quadratic sum of EMG *J* for all muscles as shown in Fig 7C, we can observe that *J* increases to a maximum around the 10th trial and then decreases in the last 10 trials. This suggests that effort optimization is a non-monotonic process. During the first 10 trials, subjects may have given priority to order selection than to optimization in the muscle activation space, which is also reflected in the behavior of *R* ^2^ values in Fig 3. Once the optimal order is selected, subjects exhibit monotonic optimization in the muscle activation space, as seen in the last 10 trials of Fig 7C, with a corresponding increase of *R* ^2^ values in Fig 3. However, our observation on the guider’s muscle activation gradually progresses from an initial muscle co-contraction based command generation strategy to a low energy policy with minimum muscle co-contraction. Therefore, this is in agreement with other studies that show a similar pattern of reduction in muscle co-contraction when motor learning progresses [24, 25]. This phenomenon can be the result of the fact from the fact that the guiding agent builds internal models [32] of hand and task dynamics to guide the blindfolded follower. The human guiding strategy can be realized by a planar 1-D of freedom robotic arm with a passive joint at the end point to connect the hard rein. We demonstrated the effectiveness of this idea by exporting with no modifications the controller identified from human-human demonstrations directly on the planar robotic arm.

### Future applications and research directions

The guiding control policy in Eqs (1) and (2) together with the virtual damped inertial model which estimates the trust level of the follower opens up the opportunity for the development of an integrated controller that treats the trust level of the follower as a part of the state vector. This will enable the controller to adjust to the changes of the behavioral dynamics of the follower in varying distraction and stress conditions. In this study we propose a model that predicts the future states of the follower and can be used in a predictive control policy. This forward model may contain some approximation of the follower’s reactive behavior. It will be interesting to understand the detailed computational nature of this prediction used by the guider. Another unexplored area is to understand factors determining how the follower perceives haptic control commands given by the guider.

Moreover, If a group can be trained to follow the person immediately in front or a leader, the robot can guide just one human using a hard rein, and that person can be linked to the others using hard or soft reins. Therefore, the group can be modeled as a soft passive dynamic system with multiple degrees of freedom.

## Materials and Methods

We conducted three separate experiments: 1) understand state dependent control policy of human subjects when one human guides another human with limited visual and auditory environmental perceptions in an arbitrary complex path, 2) model the trust level of the follower using a time varying damped inertial system, and 3) validate the guider’s control policy.

### Experimental protocol

In all experiments, subjects signed a written consent form approved by King’s College London Bio medical Sciences, Medicine, Dentistry and Natural and Mathematical Sciences research ethics committee which approved this study by Kings College London Bio medical Sciences, Medicine, Dentistry and Natural and Mathematical Sciences research ethics committee (REC Reference number BDM/11/12-20.).

### Experiment 1: Extracting guiding/following control policies

Experiment 1 was conducted to extract guiding/following control policies. Fifteen (11 male, 4 female) naive subjects participated in 20 trials. Subjects were healthy and in the 23—43 age group (avg: 28.20, std: 5.12) years. Fig 1A shows how the guider and the follower held both ends of a hard 0.7m long, 500g weight rein to track the wiggly path. Fig 1A shows the follower was blindfolded and cutoff and prevented from using auditory feedback. Fig 1B shows the relative orientation difference between the guider and the follower (referred to as state hereafter), and angle of the rein relative to the guiding agent (referred to as action hereafter).

For clarity, the detailed wiggly path is shown in Fig 1C. The 9m length path was divided into nine milestones as shown in Fig 1C. In any given trial, the guider was asked to take the follower from one milestone to another at six milestones up or down (ex. 1–7, 2–8, 3–9, 9–3, 8–2, and 7–1). The starting milestone was pseudo-randomly changed from trial to trial and the follower was disoriented before starting every trial in order to eliminate the effect of any memory of the path. The guider was instructed to move the handle of the hard rein only on the horizontal plane to generate left/right turn and push/pull commands. In that scenario, the guider takes only left/right and push/pull movements in horizontal plane with negligible vertical movements. Furthermore, the guider was instructed to use push and pull commands for forwards and backwards movements to track the follower in defined path as shown in Fig 1C. The follower was instructed to pay attention to the commands via the hard rein to follow the guider. The follower started to follow the guider once a gentle tug was given via the rein. The subjects were asked to maintain a natural speed of walking during the trial. Experimental data can be found: S1 File: Motion data for subject 1 to subject 8, S2 File: Motion data for subject 9 to subject 15. Moreover, S3 File: EMG data for subject 1 to subject 8, and S4 File: EMG data for subject 9 to subject 15.

### Experiment 2: Modeling the follower’s trust in different paths

Experiment 2 was conducted to study how to model the trust of the follower in different path tracking contexts. Fourteen naive pairs (10 male, 4 female) of subjects participated in 10 trials each for three different paths as shown in Fig 1E. Subjects were healthy and in the 23–43 age group (avg: 26.20, std: 2.21) years. The path was pseudo-randomly changed from trial to trial and the follower was disoriented before starting every trial in order to eliminate the effect of any memory of the path. The subjects were given 5 minute breaks after every 6 trials. Moreover, the subjects were asked to maintain a natural speed of walking during the each trial. To study the trust from the human follower, a trust scale 1 to 10 ranging from lowest to highest was introduced before starting the experiments and subjects were asked to rate their trust in following the guider after each trial. Experimental data can be found: S5 File: Force data for subject 1 to subject 5, S6 File: Force data for subject 6 to subject 10, and S7 File: Force data for subject 11 to subject 15. Moreover, S8 File: Motion data for subject 1 to subject 8, and S9 File: Motion data for subject 9 to subject 15.

### Experiment 3: Validating the guider’s control policy

Experiment 3 was conducted to validate the guider’s control policy and test its stability. We conducted experiments with 10 naive subjects (7 male, 3 female). Subjects were healthy and in the 21–28 age group (avg: 25.90, std: 1.91) years. Each subject participated in 3 trials. We implemented the guider’s control policy in Eq (7) on a 1-DoF planar robotic arm to generate swing actions to guide a follower to a desired point. The schematic diagram of the experimental setup is shown in Fig 1F. Fig 1G shows the actual experimental setup.

Here a cord was attached to the waist belt of the blindfolded subjects. The the encoder on the shaft platform is used to measure the orientation difference between the follower and the motor shaft as shown in Fig 1G. The subjects were instructed to move proportional to the force they felt and to the direction of the tug force. Once the trial was started, the encoder mounted on the motor shaft read instantaneous error *ϕ* of the blindfolded subject’s position relative to the desired angle. We defined −65°, −45°, −25°, +25°, +45°, and +65° as desired angles. Then the robotic arm computed the commands to perturb the arm to minimize the following error between the human subject and the robotic arm. A single trial was run for 90 seconds. Experimental data can be found: S10 File: Reaching data for 10 subjects.

### Sensing

MTx motion capture sensors (3-axis acceleration, 3-axis magnetic field strengths, 4-quaternions, 3-axis Gyroscope readings (Xsens,USA)) were used to measure the states *ϕ* and actions *θ* of the duo. Two MTx sensors were attached on the chest of the guider and the follower to measure the rate of change of the orientation difference between them (state). Another motion tracker was attached on the hard rein to measure the angle of the rein relative to the sensor on the chest of the guider (action from the guider). Four Electromyography (EMG) electrodes at 1500Hz were fixed on the guider’s anterior deltoid, biceps, posterior deltoid and lateral triceps along the upper arm as shown in Fig 6D. Before attaching EMG electrodes, the skin was cleaned with alcohol. An extra motion tracker with a switch was worn by the guider. We achieved synchronization of MTx motion sensors with muscle EMG sensors by serially connecting a channel of the EMG recorder with the magnetic sensor of the MTx sensor via a switch. The guider switched on the circuit which induced a magnetic pulse in the MTx motion sensor while recording a voltage pulse in one of the channels of the EMG records. Since we used five MTx sensors, we sampled data at 25Hz to stay within hardware design limits.

In the second experiment, in addition to MTx sensors, ATI Mini40 6-axis force torque transducer was attached to the hard rein to measure tug force sampled at 1000Hz along the horizontal plane to guide the follower. The acceleration of the follower was measured by MTx sensors as shown in Fig 1B.

### Data Analysis

All data were analyzed using MATLAB R2012a (The MathWorks Inc). We used Daubechies wave family (db10) of the MATLAB Wavelet Toolbox to extract the action of the guider and the state of the follower. Symlet wave family (sym8) of MATLAB was used for EMG analysis. Statistical significances were computed using the Mann-Whitney U test and t-test.

### Ethics statement

The experimental study, protocol, information sheet, and consent form were approved by the King’s College London Biomedical Sciences, Medicine, Dentistry and Natural and Mathematical Sciences research ethics committee: REC Reference number BDM/11/12-20. In all experiments, subjects signed a written consent form approved by King’s College London Bio medical Sciences, Medicine, Dentistry and Natural and Mathematical Sciences research ethics committee.

## Supporting Information

S1 File Motion data in human movements in Experiment 1 for subject 1 to subject 8. (ZIP)

S2 File Motion data in human movements in Experiment 1 for subject 9 to subject 15. (ZIP)

S3 File EMG recordings in Experiment 1 for subject 1 to subject 8. (ZIP)

S4 File EMG recordings in Experiment 1 for subject 9 to subject 15. (ZIP)

S5 File Force data in Experiment 2 for subject 1 to subject 5. (ZIP)

S6 File Force data in Experiment 2 for subject 6 to subject 10. (ZIP)

S7 File Force data in Experiment 2 for subject 11 to subject 15. (ZIP)

S8 File Motion data in human movements in Experiment 2 for subject 1 to subject 8. (ZIP)

S9 File Motion data in human movements in Experiment 2 for subject 9 to subject 15. (ZIP)

S10 File Reaching data in Experiment 3 for 10 subjects. (ZIP)
